# A Machine Learning Approach to Predict Successful Trans-Ventricular Off-Pump Micro-Invasive Mitral Valve Repair

**DOI:** 10.3390/jcm14165863

**Published:** 2025-08-19

**Authors:** Alessandro Vairo, Caterina Russo, Andrea Saglietto, Rino Andrea Cimino, Marco Pocar, Cristina Barbero, Andrea Costamagna, Gaetano Maria De Ferrari, Mauro Rinaldi, Stefano Salizzoni

**Affiliations:** 1 Division of Cardiology, Cardiovascular and Thoracic Department, Città della Salute e della Scienza Hospital, 10126 Turin, Italy; dottcaterinarusso92@gmail.com (C.R.); gaetanomaria.deferrari@unito.it (G.M.D.F.); 2 Department of Medical Sciences, University of Turin, 10126 Turin, Italy; mauro.rinaldi@unito.it; 3 Division of Cardiac Surgery, Department of Surgical Sciences, University of Turin, 10126 Turin, Italy; marco.pocar@unito.it (M.P.); cristina.barbero@unito.it (C.B.); stefano.salizzoni@unito.it (S.S.); 4 Division of Anaesthesia and Critical Care, Department of Surgical Sciences, Città della Salute e della Scienza Hospital, 10126 Turin, Italy; andrea.costamagna@unito.it

**Keywords:** severe mitral insufficiency, minimally invasive cardiac surgery, echocardiography, mitral valve prolapse, NeoChord device

## Abstract

**Background**: The NeoChord procedure is a trans-ventricular, echo-guided, beating-heart mitral valve (MV) repair technique used to treat degenerative mitral regurgitation (MR) caused by leaflet prolapse and/or flail. **Objectives**: This study aimed to develop a machine learning (ML) scoring system using pre-procedural clinical and echocardiographic variables to predict the success of the NeoChord procedure—defined as less than moderate MR at follow-up. **Methods:** A total of 80 patients were included. Preoperative MV anatomical parameters were assessed using three-dimensional (3D) transesophageal echocardiography and analyzed with dedicated post-processing software (QLAB software, version 15.0, Philips Healthcare, Amstelveen, NL, The Netherlands). Two supervised ML models (random forest and decision tree) were trained on the dataset, with hyperparameters optimized via 10-fold cross-validation. The random forest model also provided a variable importance ranking using a filter-based method. Key predictors identified by the models included age, flail gap, early systolic mitral valve area, and indexed left atrial volume. **Results**: The mean and median cross-validated area under the curve of the ML models were 0.79 and 0.83 for the random forest model and 0.72 and 0.77 for the decision tree model, respectively. **Conclusions**: A machine learning approach integrating clinical and 3D echocardiographic parameters can effectively predict mid-term procedural success of the NeoChord technique. This method may support future preoperative patient selection, pending validation in larger cohorts.

## 1. Introduction

Degenerative mitral regurgitation (MR) is one of the most common valvular heart diseases requiring surgical intervention in Western countries [1,2,3]. In recent years, multiple techniques and devices have emerged to enable mitral valve repair (MVR) without the need for cardiopulmonary bypass, thereby reducing perioperative risks [4]. Among these, trans-ventricular beating-heart mitral valve repair (TBMVR) with artificial chordae implantation has proven to be both feasible and safe [5,6]. This echo-guided, off-pump procedure has demonstrated promising mid-term clinical outcomes [7]; however, patient selection remains a critical determinant of long-term durability [8,9]. Three-dimensional (3D) transesophageal echocardiography plays a central role in guiding the NeoChord procedure, both for optimal positioning of the neochordae and for real-time morphological assessment during traction and fixation [10]. The anatomical complexity of mitral valve disease strongly influences procedural success [11,12]. A commonly used classification identifies four types of pathology [12], with type A (isolated central posterior leaflet prolapse) being most suitable for the NeoChord technique due to the straightforward access via the ventricular route [8]. More complex anatomies—such as multisegment posterior leaflet prolapse or isolated non-central prolapse (type B), anterior or bileaflet prolapse (type C), and paracommissural prolapse (type D)—are associated with less favorable outcomes, even with conventional surgical repair [9]. Due to the absence of standardized selection criteria, Colli et al. proposed the leaflet-to-annulus index (LAI) as a key echocardiographic metric to evaluate NeoChord feasibility: LAI is calculated as the ratio of the combined anterior and posterior leaflet heights to the anteroposterior annular diameter [13]. A threshold value of 1.25 indicates sufficient leaflet tissue relative to annular size, correlating with a higher likelihood of achieving mild or less MR at 1-year follow-up [13]. Although annular dilation can compromise outcomes, LAI offers patient-specific insight: if adequate leaflet tissue is present, the NeoChord procedure may still be viable despite some degree of annular enlargement [14]. Additional predictive tools have recently been developed. Manzan et al. introduced a nomogram in 2021 incorporating seven clinical and echocardiographic variables to estimate the likelihood of mild or less MR at long-term follow-up [15]. Moreover, Vairo et al. demonstrated the predictive potential of static and dynamic 3D mitral annulus measurements for successful MV repair without the need for reductive annuloplasty [16]. In light of the increasing need for precise, individualized patient selection in off-pump mitral repair, we aimed to evaluate whether artificial intelligence (AI) techniques—specifically machine learning—could identify key preoperative echocardiographic predictors of procedural success in the NeoChord procedure.

## 2. Materials and Methods

### 2.1. Patient Cohort and Procedure Indication

The aim of this present study was to develop a machine learning (ML)-based scoring system using pre-procedural clinical and echocardiographic variables to predict the success of the NeoChord procedure at 3-year follow up. Procedural success was defined as residual MR less than moderate.

This retrospective analysis included 80 consecutive patients who underwent mitral valve repair using the NeoChord DS1000 system (NeoChord, Inc., Eden Prairie, MN, USA) between March 2015 and April 2023 at the Città della Salute e della Scienza Hospital in Turin, Italy. All patients had severe degenerative mitral regurgitation due to prolapse and/or flail. Indications for the procedure were determined by the amultidisciplinary Heart Team based on clinical, anatomical, and patient-specific factors. These included contraindications to conventional surgery with cardiopulmonary bypass—such as high surgical risk, significant comorbidities, or frailty—as well as the patient preference for a micro-invasive approach.

### 2.2. Inclusion and Exclusion Criteria

Patients were eligible for inclusion if they had anatomical features suitable for mitral valve repair using the NeoChord technique. Exclusion criteria included functional MR, congenital mitral valve anomalies, or active endocarditis.

Patients who died from unrelated causes or who had not yet completed three years of follow-up were also excluded. Specifically,

- Six patients died from non-cardiac causes (e.g., cancer, leukemia, respiratory failure);
- Eleven patients underwent mitral valve reoperation due to recurrent severe MR;
- Twenty-nine patients had not yet reached the 3-year follow-up mark, as they underwent the procedure more recently.

Anatomical suitability was assessed through transthoracic and transesophageal echocardiography using iE33 or Epiq Cvx systems (Philips Healthcare, Amstelveen, NL, The Netherlands). Mitral valve morphology was classified into the following categories:

- Type A: Isolated central posterior mitral leaflet disease.
- Type B: Lateral, medial, or multi-segment posterior mitral leaflet disease.
- Type C: Anterior mitral leaflet disease or bi-leaflet involvement.

No patient with Type D underwent the NeoChord procedeure.

Patients with Type C morphology or other factors controindicating cardiopulmonary bypass were considered compassionate cases.

Only patients with Type II Carpentier MR and an LAI > 1.2 were included. Standard 2D echocardiographic measurements were performed along with detailed assessments of mitral annulus dimensions (anteroposterior and intercommissural diameters), anterior and posterior leaflet heights, and LAI. Advanced 3D echocardiographic analysis was conducted using QLAB software (version 15.0, Philips Healthcare, Amstelveen, NL, The Netherlands), allowing quantification of mitral annulus area, leaflet area, and regurgitant orifice, along with systolic, diastolic, and early-systolic measurements.

Postoperative follow-up was conducted at discharge and at 1–3 months, 6 months, and 12 months, followed by annual evaluations. MR severity was graded according to the American Society of Echocardiography criteria. Clinical outcomes were assessed following the Mitral Valve Academic Research Consortium (MVARC) guidelines. The primary endpoint was procedural success, defined as freedom from moderate or severe MR; no rehospitalization or reintervention; improvement in New York Heart Association (NYHA) functional class.

### 2.3. Predictive Variables and Data Collection

A total of 44 variables were collected for analysis, comprising 14 clinical and 30 echocardiographic parameters.

Clinical variables included

- Age;
- Gender;
- Comorbidities: hypertension, diabetes, dyslipidemia, coronary artery disease, atrial fibrillation;
- Smoking status;
- Surgical risk scores: EuroSCORE II and STS.

Echocardiographic variables included

- Mitral valve anatomical classification (Types A, B, C);
- Scallop involvement;
- Presence of clefts;
- Left ventricular ejection fraction (LVEF, %);
- Indexed left atrial volume (LAVi, mL/m^2^);
- Left ventricular end-diastolic diameter (LVEDD, mm);
- Left ventricular end-diastolic volume (LVEDV, mL);
- Left ventricular end-systolic volume (LVESV, mL);
- Tricuspid regurgitation (TR);
- Systolic pulmonary artery pressure (PAPs, mmHg);
- Mitral annular calcification;
- Flail gap and flail width;
- Prolapse/flail area;
- Anterior (LAM) and posterior (LPM) leaflet heights;
- Total leaflet area;
- Anteroposterior (AP) and intercommissural (IC) annular diameters (2D and 3D);
- Leaflet-to-annulus index (LAI) in 2D and 3D;
- End-systolic, early-systolic, and end-diastolic annular areas;
- End-systolic annular height;
- Annular circumference at end-systole;
- Annular area fractional change (%);
- Annular circumference fractional change (%).

All variables were grouped into two datasets:

- A general dataset, including clinical and standard echocardiographic variables;
- A morphological dataset, focused on dynamic 3D annular measurements.

Descriptive statistics were applied to summarize the data. Categorical variables were reported as frequencies and percentages, while continuous variables were presented as means ± standard deviations. All variables were tested for association with procedural outcomes using appropriate statistical methods.

### 2.4. Data Analysis: Statistical Methods and Machine Learning Approaches

Statistical analysis was performed using the Statistical Package for Social Sciences (SPSS, version 29.0.2.0, Inc., Chicago, IL, USA). Univariate analysis was used to assess the associations between clinical and echocardiographic variables and procedural outcomes. Continuous variables were analyzed with Student’s *T*-test, and categorical variables with the chi-square test (Χ^2^). A *p*-value < 0.05 was considered statistically significant. Given the relatively small sample size, machine learning (ML) models were employed to develop predictive models for procedural success. Two supervised classification algorithms were used: random forest (RF) and decision tree (DT), both trained on the full dataset. Missing data were handled using the k-nearest neighbors (kNN) imputation with K = 5. Only two variables with less than 50% missing values were imputed; those with greater missingness were excluded.

To maximize data utilization and ensure model generalizability, a 10-fold cross-validation was used during training. Model performance was assessed by calculating the area under the curve (AUC):

- AUC ≤ 0.6: poor.
- AUC ≈ 0.8: good.
- AUC = 1.0: excellent.

Before model training, a feature selection process was performed using the Boruta algorithm, a wrapper method based on the random forest classifier. Boruta identifies relevant features by comparing the importance of real variables to randomized shadow features, retaining only those with statistically significant contributions. Only variables selected by Boruta were used to train the final models.

Hyperparameter tuning for both RF and DT classifiers was performed using grid search with internal cross-validation, implemented through the caret package in R (version 4.0.0) to reduce overfitting and improve generalizability.

In addition to global model performance, the random forest model was used to assess variable importance, ranking features by their relative contribution to predictive accuracy using a filter-based approach.

All machine learning analyses were conducted in R (version 4.0.0) using the caret package.

## 3. Results

Overall, all 80 patients included in the registry were analyzed. Baseline clinical and echocardiographic characteristics are summarized in Table 1 and Table 2.

The mean age was 77 ± 9.2 years, and 35% were female. One in four patients had a history of atrial fibrillation. Eleven patients (13.7%) had previously undergone cardiac surgery, including eight with prior mitral valve repair. All patients had degenerative MVP, with predominantly favorable anatomy (Type A or B in 86.2% of cases). The left atrium was typically dilated preoperatively, with a mean indexed left atrial volume (LAVi) of 61 mL/m^2^. The mean indexed left ventricular volume was 71 mL/m^2^, and the average left ventricular ejection fraction (LVEF) was 63%. Nineteen patients had an estimated systolic pulmonary artery pressure (PAPs) > 50 mmHg, and 18 (22.5%) had moderate or severe tricuspid regurgitation.

A median of three neochordae were implanted per procedure. Six patients presented with at least moderate MR at discharge, including two with moderate-to severe: in one case only one NeoChord was implanted due to unfavorable anatomy; in the other, a native chordae ruptured a few days after the procedure.

Follow-up assessments were completed in 94% at 3 months, 86% at 6 months and 1 year, 73% at 2 years, and 59% at 3 years, excluding patients who died or required surgical reintervention due to recurrent MR. At 3-year follow-up, 30 patients (37.5%) developed recurrent MR of at least moderate severity. Procedural and midterm outcomes are comparable to the largest series published to date, including those from the Padua and Vilnius centers, which also reported 3-year follow-up results. Table 3 and Table 4 present baseline clinical and echocardiographic parameters, stratified by the presence of a significant MR during follow-up.

Patients with moderate or grater recurrent MR were more likely to have a history of atrial fibrillation and arterial hypertension. A more severe target lesion—defined by a larger flail gap—was associated with higher failure. As previously shown by Vairo et al., patients with recurrent MR had greater LA volumes and had more dilatated and dysfunctional mitral annulus, with lower coaptation reserve indexes than those with durable repair. Importantly, this analysis confirmed that dynamic annular dysfunction, particularly involving early systolic annular area and circumference measured in three-dimensional imaging, was strongly associated with procedural success. Patients with smaller preoperative annular areas and circumferences during early systole were significantly more likely to maintain less-than-moderate MR at follow-up.

### 3.1. Feature Selection for ML Models

**Figure 1:**
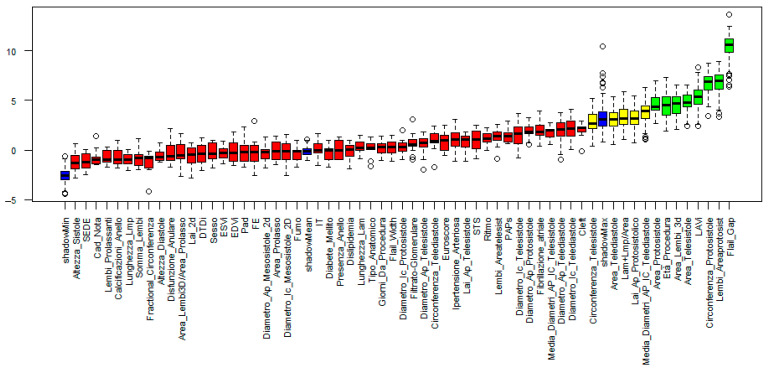
Feature selection results obtained using the Boruta algorithm (full variable set). Variables are ranked based on their importance in the classification task. Green bars indicate features confirmed as relevant, yellow bars represent tentative features, and red bars denote features rejected as non-informative for predicting the outcome.

**Figure 2:**
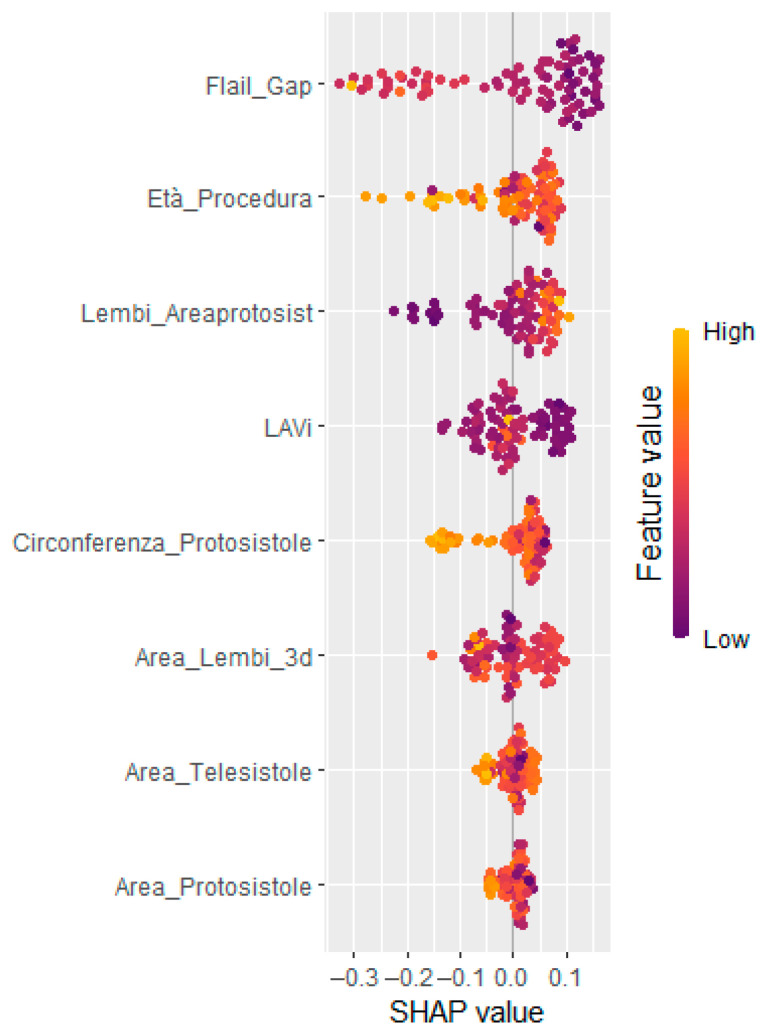
SHAP summary (beeswarm) plots showing the most relevant clinical and echocardiographic contributing to model output. Each dot represents a single observation; feature values are color-coded (purple = low, yellow = high). Features are ordered by their average absolute SHAP value.

Before model training, missing data were imputed as described in the Section 2. We then applied the Boruta algorithm to identify the most informative variables for predicting procedural outcomes. Feature selection was performed on two different sets of input variables:

- In the full variable set (Figure 1), which included both clinical and echocardiographic parameters, the following features were retained: Procedural Age, Left Atrial Volume Index (LAVi), Flail Gap, Protosystolic Area, Telesystolic Area, Protosystolic Circumference, 3D Leaflet Area, and Leaflet Protosystolic Area.
- In the reduced set (Figure 2), which included only echocardiographic variables, the Boruta algorithm identified the following: Left Atrial Volume Index (LAVi), Flail Gap, Telesystolic Area, Protosystolic Circumference, 3D Leaflet Area, and Leaflet Protosystolic Area.

Drawing upon these findings, machine learning models were developed and concurrently evaluated using both sets of selected features.

### 3.2. ML Models Based on Both Clinical and Echocardiographic Parameters

Using the set of variables identified through the Boruta algorithm (see Section 3.1), random forest (RF) and decision tree (DT) classifiers were developed and validated with a 10-fold cross-validation.

The RF model demonstrated superior performance, with a median AUC of 0.90 and a mean AUC of 0.83 across resamples. The DT model achieved a median AUC of 0.78 and a mean of 0.74, indicating moderately lower accuracy.

**Figure 3:**
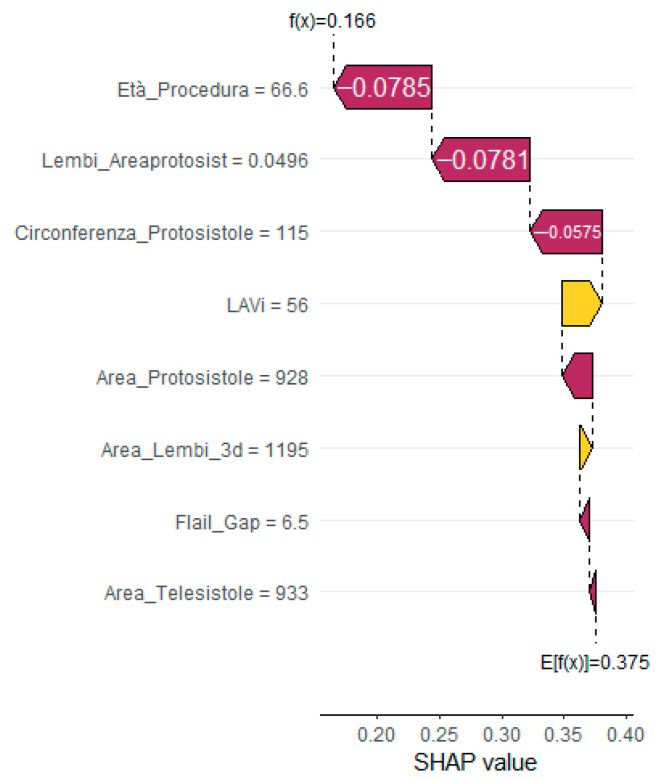
SHAP force plot for a representative patient without recurrent MR, visualizing the direction and magnitude of each feature’s contribution to the predicted outcome. Yellow bars indicate positive contributions (increased risk); purple bars indicate negative contributions (reduced risk).

To enhance interpretability and support clinical decision making, SHapley Additive exPlanations (SHAP) analysis was applied. The global SHAP summary plot (Figure 2) revealed that Flail Gap, age at the time of procedure, and indexed left atrial volume (LAVi) were the most influential predictors of recurrent mitral regurgitation. Additionally, SHAP force plots (Figure 3) provided individualized explanations of model predictions, highlighting patient-specific risk profiles.

### 3.3. ML Models Based on Echocardiographic Parameters Only

To assess the predictive value of imaging data independently, RF and DT models were also trained using only echocardiographic variables selected by the Boruta algorithm.

**Figure 4:**
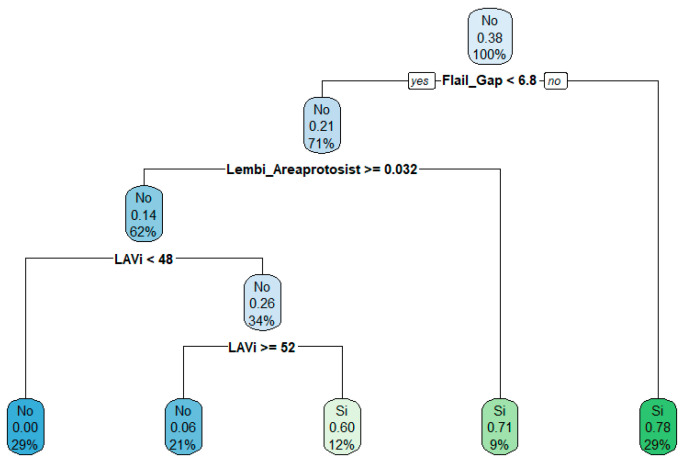
Decision tree derived from echocardiographic parameters. The tree displays the most relevant decision rules, with terminal nodes indicating the predicted class, associated probability, and distribution of observations. Node colors reflect prediction confidence.

The RF model maintained a median AUC of 0.90 and mean AUC of 0.82, indicating performance comparable to the combined-variable model. SHAP analysis confirmed that Flail Gap, LAVi, and end-systolic annular area were among the most influential echocardiographic predictors (Figure 4).

The DT model built on echocardiographic data alone showed a median AUC of 0.77 and a mean AUC of 0.72, confirming a moderate but acceptable performance.

These findings indicate that echocardiographic parameters alone can provide robust predictive accuracy for identifying patients at risk of recurrent mitral regurgitation, underlining the prognostic value of advanced mitral valve imaging.

## 4. Discussion

Trans-ventricular, off-pump, beating-heart neochordae implantation is a microinvasive surgical approach for the treatment of severe primary MR. Within this evolving field, both accurate patient selection and heart team experience have emerged as key determinants of procedural success [10]. Previous studies have highlighted the significance of the surgeon’s learning curve, with the final “expert phase” reached after approximately 50 procedures [8,17]. Additionally, coordinated teamwork and effective communication between the surgeon and echocardiographer are essential to ensure optimal procedural planning and intraprocedural imaging guidance.

Over time, the ability to appropriately select patients for this procedure has improved. Initially, anatomical classification of the mitral lesion served as the primary criterion for eligibility [18]. However, the introduction of the leaflet-to-annulus index (LAI) marked a significant advancement, quantifying the excess leaflet tissue in relation to annular dimensions and correlating with the predicted coaptation surface post-repair [13]. Three-dimensional (3D) echocardiographic assessment has since proven superior to two-dimensional (2D) analysis in calculating this index and in evaluating overall mitral apparatus morphology [16].

The first echocardiographic nomogram to support patient selection for NeoChord implantation was developed by the Padova group, incorporating seven variables to predict procedural success [15]. Building upon this approach, the present study employed machine learning (ML) algorithms to develop a predictive tool capable of estimating the risk of recurrent MR. Artificial intelligence, particularly ML, is a field undergoing swift development, finding growing application within cardiac healthcare. Indeed, contemporary investigations have demonstrated that AI-driven models for cardiovascular risk prediction surpass conventional risk assessment tools [19].

ML offers distinct advantages over traditional statistical methods by modeling complex, non-linear interactions among clinical and echocardiographic variables without the need to manually specify interaction terms [20].

The resulting decision-support model emphasized a multiparametric evaluation strategy. Among clinical predictors, patient age was found to have the highest impact on procedural outcome. Aging is known to induce atrial myocardial fibrosis, which predisposes to atrial remodeling and atrial fibrillation—factors associated with negative outcomes in mitral valve repair [21,22,23,24]. Recent proposals have suggested that preoperative assessment of left atrial fibrosis in asymptomatic patients with primary MR may aid in optimizing surgical timing [25].

Anatomical variables also played a critical role. Flail gap emerged as a strong predictor, reflecting the severity of the target lesion and its influence on leaflet grasping feasibility. Left atrial volume index (LAVi), a well-established indicator of MR severity and cardiac remodeling, demonstrated independent predictive value [17,25]. Therefore, it represents a predictor variable of MVr with NeoChord success because it is indirectly associated with annular dimension and has itself a role in the progression of the annular disfunction, causing relapse on follow-up. Although a previous report indicated that an LAI > 1.25 measured via 2D imaging predicted favorable outcomes, this study found that LAI maintained statistical significance only when derived from 3D anterior–posterior annular diameter measurements—further confirming the superior prognostic utility of 3D assessment [16].

Among time-varying 3D annular parameters, early systolic annular area and circumference were most strongly associated with favorable outcomes. This is consistent with previous evidence suggesting that early systole represents the phase of maximal annular contraction, during which the most pronounced differences in annular geometry between normal and pathological valves are observed [26]. Prior investigations using both in vivo 3D echocardiography and ex vivo simulators have demonstrated that the dynamic contraction of the mitral annulus during early systole is critical for proper or proper leaflet coaptation, reducing posterior leaflet stress and overall systolic loading [26,27]. In healthy and degenerative MR patients, early systolic annular narrowing is typically followed by gradual expansion; however, in dysfunctional annuli (e.g., ischemic or myxomatous valves, or those associated with atrial fibrillation), this dynamic response is attenuated, with a significantly reduced annular fractional change (19% vs. 10%) [28]. Overall, these findings reinforce the prognostic value of integrating clinical and advanced echocardiographic parameters—particularly dynamic 3D annular measurements—into the selection process for transventricular neochordal mitral valve repair. The adoption of ML-based tools may support more precise, individualized patient selection and ultimately improve long-term procedural outcomes.

## 5. Limitation

The primary limitation of this study lies in the relatively small sample size, which precluded the possibility of setting aside a dedicated test set for model evaluation. Furthermore, external validation using data from other institutions was not performed, thus partially limiting the generalizability of the findings. Nevertheless, the application of a 10-fold cross-validation strategy provided robust cross-validated AUC estimates, which are widely considered reliable proxies for out-of-sample model performance. Another important factor to consider is the procedural learning curve associated with this novel technique. The inclusion of early cases, performed during the initial phase of the learning curve, may have influenced overall outcomes, potentially leading to suboptimal results in this subgroup. Additionally, the overall success rate may have been influenced by a degree of selection bias. In several cases, the procedure was offered on a compassionate basis to high-risk patients for whom no alternative surgical options were deemed feasible. These patients often presented with unfavorable prognoses and/or anatomical characteristics less suitable for the procedure. As a result of these limitations, the statistical inference drawn from the current dataset should be interpreted with caution and regarded primarily as a foundation for further prospective, multicenter investigations involving larger, more diverse populations.

## 6. Conclusions

Trans-ventricular off-pump beating-heart neochordae implantation is a microinvasive cardiac surgery procedure indicated for patient suffering from degenerative MR. In carefully selected patients, this procedure has demonstrated favorable outcomes at follow-up, particularly in terms of MR recurrence. However, strict and structured patient selection remains essential for procedural success.

Over time, numerous parameters have been proposed to aid preoperative assessment, but a comprehensive and standardized selection strategy has yet to be established. The decision algorithm proposed in this study offers an objective, multiparametric framework—integrating clinical variables and advanced three-dimensional echocardiographic analysis—to support the selection of suitable candidates for NeoChord procedure. While predictive models are not a substitute for clinical expertise or patient preference, they can serve as valuable adjuncts in decision making. A systematic, multiparametric preoperative evaluation—supported by validated predictive tools—should become standard practice when selecting candidates for NeoChord mitral valve repair.

## Figures and Tables

**Table 1:** Baseline demographic and clinical patient’s characteristics.

Parameter	Mean ± SD
Age (years)	77 ± 9.2
eGFR (mL/min)	63.6 ± 22.6
Euroscore II (%)	2.3 ± 1.85
**Parameter**	**N%**
Female	77 ± 9.2
NYHA class II or IV	63.6 ± 22.6
DM II	2.3 ± 1.85
Arterial hypertension	50 (62.5%)
Extracardiac arteriopathy	7 (8.7%)
CAD	12 (15%)
Previous stroke	2 (2.5%)
COPD	5 (6.3%)
Malignancy	14 (17.5%)
AF	24 (30%)
Persistent AF	13 (16.3%)
Previous cardiac surgery	11 (13.7%)
PAPs > 50 mmHg	19 (23.7%)
Compassionate	23 (28.7%)

eGFR: estimated glomerular filtration rate; NYHA: New York Heart Association classification; CAD: coronary artery disease; COPD: chronic obstructive pulmonary disease; AF: atrial fibrillation; PAPs: systolic pulmonary artery pressure.

**Table 2:** Baseline echocardiographic parameters.

Parameter	Mean ± SD
EF (%)	63 ± 5
LVEDD (mm)	53 ± 7
EDVi (mL/m^2^)	71 ± 21
ESVi (mL/m^2^)	26 ± 7
LAVi (mL/m^2^)	61 ± 25
sPAP (mmHg)	43 ± 15
AP annulus diameter 2D (mm)	31.1 ± 5.6
IC annulus diameter 2D (mm)	36.2 ± 5.7
AP annulus diameter 3D (mm)	34.4 ± 5.9
IC annulus diameter 3D (mm)	42.6 ± 5.8
AML (mm)	26.6 ± 4
PML (mm)	17.7 ± 2.9
LAI (2D)	1.45 ± 0.24
LAI (3D)	1.42 ± 0.06
Flail gap (mm)	5.9 ± 2.1
Flail width (mm)	13.6 ± 4.6
Prolapse area (mm^2^)	209 ± 90
Annulus area, end-systolic (mm^2^)	1197.8 ± 312.7
Annulus area, early-systolic (mm^2^)	1120.5 ± 326.1
Annulus area, end-diastolic (mm^2^)	1294.1 ± 334
Annulus circumference, end-systolic (mm)	134 ± 17.1
Annulus area fractional chance (%)	7.6 ± 6.1
Annulus circumference fractional change (%)	4.4 ± 3.3
Leaflets area (mm^2^)	1553 ± 390
Annulus heigh, end-systolic (mm)	7.6 ± 2.4
**Parameter**	**N (%)**
TR ≥ moderate	18 (22.5%)
Anatomical Classification	
Type A (favorable)	50 (62.5%)
Type B (favorable)	19 (23.7%)
Type C (unfavorable)	11 (13.8%)
Spot annulus calcification	8 (10%)
Cleft	4 (5%)

EF: ejection fraction (%); LVEDD: left ventricular end-diastolic diameter; EDVi: left ventricular end-diastolic volume indexed; ESVi: left ventricular end—systolic volume indexed; LAVi: indexed left atrial volume; sPAP: systolic pulmonary artery pressure; AP: antero-posterior; IC: inter-commissural; AML: anterior mitral leaflet; PML: posterior mitral leaflet; LAI: leaflet to-annulus index; TR: tricuspidalic regurgitation.

**Table 3:** Baseline clinical variables, stratified by MR recurrence during follow-up.

Parameter	Success Group n = 50	Failed Group n = 30	OR	Univariate 95% CI	Logistic Regression *p*-Value
Age (years)	74 ± 9	77 ± 10	1.05	0.99–1.11	0.071
Euroscore II (%)	2.3 ± 1.9	2.8 ± 1.9	1.14	0.90–1.45	0.268
eGFR (mL/min)	67.2 ± 24.5	57.8 ± 19.8	0.98	0.95–1.00	0.077
Female	15 (30%)	10 (33%)	0.97	0.37–2.53	0.951
DM II	3 (6%)	1 (3%)	0.54	0.05–5.44	0.601
Arterial hypertension	27 (54%)	24 (80%)	3.40	1.118–9.77	0.023
Extracardiac arteriopathy	3 (6%)	4 (13%)	2.41	0.50–11.60	0.273
CAD	9 (18%)	5 (17%)	0.91	0.27–3.02	0.879
Atrial fibrillation	9 (18%)	15 (50%)	4.55	1.64–12.58	0.003
Previous cardiac surgery	8 (16%)	3 (10%)	0.82	0.14–4.78	0.827

eGFR: estimated glomerular filtration rate; DM II: type II diabetes mellitus; CAD: coronary artery disease.

**Table 4:** Baseline echocardiographic variables, stratified by MR recurrence during follow-up.

Parameter	Success Group n = 50	Failed Group n = 30	*p*-Value
Ejection fraction (%)	63.2 ± 4.6	62.6 ± 5.8	0.69
LVEDD (mm)	30.2 ± 0.9	30.7 ± 1.1	0.74
EDVi (mL/m^2^)	69.9 ± 3.2	74 ± 3.8	0.42
ESVi (mL/m^2^)	25.7 ± 1.1	28.8 ± 1.5	0.15
LAVi (mL/m^2^)	52.7 ± 2.5	67.2 ± 5.5	0.01
sPAP (mmHg)	27 (54%)	24 (80%)	0.11
Prolapse area (mmq)	2.02 ± 0.14	2.23 ± 0.21	0.408
AML + PML (mm)	43.85 ± 0.83	44.87 ± 0.64	0.243
Leaflet area 3D (mm^2^)	1397.81 ± 49.25	1687.59 ± 81.58	0.001
Leaflet area/prolapse area	793.36 ± 63.52	927.08 ± 110.42	0.267
Flail gap (mm)	5.03 ± 0.24	7.14 ± 0.42	0.001
Flail width (mm)	12.95 ± 0.91	15.16 ± 1.46	0.186
Cleft	0	4 (13%)	0.017
Anulus, static			
AP annulus diameter 2D (mm)	30.52 ± 0.84	32.18 ± 0.94	0.22
End-systolic annulus height (mm)	7.99 ± 0.40	7.04 ± 0.39	0.003
IC annulus diameter 2D (mm)	35.81 ± 0.78	36.55 ± 1.16	0.62
AP annulus diameter, early-systolic, 3D (mm)	30.2 ± 5.18	34.9 ± 4.53	0.0005
AP annulus diameter, end-systolic, 3D (mm)	32.3 ± 6.52	37.4 ± 5.92	0.0003
AP annulus diameter, end-diastolic, 3D (mm)	32.7 ± 5.79	37.5 ± 4.98	0.0005
IC annulus diameter, early-systolic, 3D (mm)	38 ± 4.81	41.4 ± 4.83	0.006
IC annulus diameter, end-systolic, 3D (mm)	39.5 ± 6.58	44.2 ± 4.91	0.0005
IC annulus diameter, end-diastolic, 3D (mm)	40.1 ± 5.81	45.1 ± 5.22	0.0022
(AP + IC)/2 3D value	36.38 ± 0.97	41.27 ± 0.95	0.0010
End-diastolic annulus area (mm^2^)	1190.39 ± 46.77	1451.48 ± 62.89	0.0001
End-diastolic annulus circumference (mm)	130.23 ± 2.68	141.97 ± 2.83	0.0001
End-systolic annulus circumference (mm)	124.33 ± 2.44	135.71 ± 2.85	0.0039
End-systolic annulus area (mm^2^)	1102.84 ± 44.97	1342.06 ± 57.30	0.0015
Annulus, dynamic			
Annulus circumference fractional change (%)	0.044 ± 0.005	0.044 ± 0.059	0.9973
Annulus area, fractional change (%)	0.076 ± 0.009	0.074 ± 0.010	0.9202
Early-systolic annulus area (mm^2^)	992.69 ± 41.43	1089.38 ± 62.25	0.0001
Early-systolic annulus circumference (mm)	119.82 ± 2.36	136.43 ± 3.02	0.0001
Coptation reserve			
Leaflet-to-annulus index (LAI) 2D	1.47 ± 0.04	1.41 ± 0.04	0.367
LAI (end-systolic AP diameter) 3D	1.40 ± 0.04	1.27 ± 0.03	0.021
LAI (early systolic AP diameter) 3D	1.50 ± 0.04	1.29 ± 0.03	0.001
(AML + PML)/annulus area (mm/mm^2^)	0.032 ± 0.001	0.027 ± 0.001	0.011

LVEDD: left ventricular end-diastolic diameter; EDVi: left ventricular end-diastolic volume indexed; ESVi: left ventricular end—systolic volume indexed; LAVi: indexed left atrial volume; sPAP: systolic pulmonary artery pressure; AML + PML: anterior mitral leaflet + posterior mitral leaflet; AP: antero-posterior; IC: inter-commissural.

## Data Availability

The study data will be made available upon request to the corresponding authors.
